# Emerging Antifungal Resistance in Dermatophytosis: A Clinicomycological Study From North India

**DOI:** 10.7759/cureus.92679

**Published:** 2025-09-18

**Authors:** Anushka Lohariwala, Sanjeev Gupta, Narinder Kaur, Aneet Mahendra

**Affiliations:** 1 Dermatology, Maharishi Markandeshwar Institute of Medical Sciences and Research, Maharishi Markandeshwar (Deemed to be University), Ambala, IND; 2 Microbiology, Maharishi Markandeshwar Institute of Medical Sciences and Research, Maharishi Markandeshwar (Deemed to be University), Ambala, IND

**Keywords:** antifungal resistance, chronic dermatophytosis, clinical dermatology, koh, tinea infections

## Abstract

Introduction

Dermatophytosis is a common superficial fungal infection affecting the skin, hair, and nails and is increasingly being recognized as a public health challenge. In recent years, there has been a noticeable increase in chronic and recurrent cases, possibly due to changing patterns of antifungal resistance, misuse of medications, and host-related factors. This study aimed to assess the demographic and clinical characteristics of patients with dermatophytosis, evaluate treatment history and recurrence, and analyze antifungal resistance patterns.

Material and methods

A total of 130 patients clinically diagnosed with dermatophytosis and attending the Dermatology Outpatient Department were included in this observational study. After obtaining informed consent, detailed data on demographic factors, clinical presentation using the Dermatophytosis Severity Score (DSS), treatment history, and recurrence were recorded. Fungal cultures and antifungal susceptibility testing were performed on positive isolates to evaluate resistance patterns.

Results

The most affected age group was 31-45 years (38; 42.2%), with a male predominance (52; 57.8%). Most patients were rural residents (72; 80%) and uneducated (58; 64.4%), with homemakers (32; 35.6%) and students (19; 21.1%) forming the major occupational groups. Disease duration was mostly one to two years (40; 44.4%), and recurrent infections were common (83; 92.2%), even with mild severity scores (DSS 1-10 in 79 individuals; 87.8%). *Tinea corporis* (79; 87.8%) was the most frequent clinical type. Poor hygiene correlated with higher DSS. Potassium hydroxide mount was positive in 115 (88.5%) patients, with culture showing *Trichophyton mentagrophytes *(87; 75.7%) as the predominant species. Resistance was seen to fluconazole (77; 85.6%) and terbinafine (54; 60%), while itraconazole, ketoconazole, and griseofulvin were sensitive.

Conclusion

Dermatophytosis continues to be a significant public health concern due to its chronicity and recurrence. Irrational use of medications and emerging resistance to commonly used antifungals highlight the need for better treatment compliance and periodic monitoring of resistance patterns.

## Introduction

Dermatophytosis refers to superficial fungal infections of keratinized tissues - skin, hair, and nails - caused by dermatophytes belonging to the genera *Trichophyton*, *Epidermophyton*, and *Microsporum* [1]. These fungi are classified into anthropophilic, zoophilic, and geophilic species based on their ecological niche. Clinical manifestations, including pruritus, erythema, and scaling, often cause significant physical discomfort and psychosocial stress [1]. Chronic dermatophytosis refers to infection persisting for more than six months to one year despite adequate treatment. Recurrent dermatophytosis is characterized by reoccurrence of lesions within a few weeks (usually less than six) after completion of treatment. Recalcitrant dermatophytosis refers to lack of clinical cure even after taking proper treatment including proper systemic antifungals for the recommended duration.

In India, the prevalence of dermatophytosis has risen sharply, accompanied by an alarming increase in chronic, recurrent, and treatment-resistant cases. Host-related risk factors such as diabetes mellitus, occlusive clothing, poor hygiene, and immunosuppression contribute to infection susceptibility. Host defense against dermatophytosis includes the skin’s physical and chemical barriers, ultraviolet exposure, and innate immunity involving keratinocytes, dendritic cells, and neutrophils. Pattern recognition receptors such as toll-like receptors (TLRs) and C-type lectin receptors (CLRs) detect fungal pattern recognition receptors (PAMPs), triggering cytokines (interleukin [IL]-6, IL-10, IL-17, TNF-α) and adaptive responses [2]. Cell-mediated immunity, especially Th1 and Th17, plays a central role in fungal clearance through IFN-γ and IL-17 [3]. In contrast, Th2 responses (IL-4, IL-5, IL-13) promote chronicity via IgE and IgG4 [4]. IL-10 suppresses Th1 but regulates immunity. Acute dermatophytosis involves delayed hypersensitivity, while chronic cases are linked to inadequate cellular responses and Th2 dominance and sometimes associated with allergic conditions such as asthma [5].

Standard treatments include topical and systemic antifungals such as terbinafine, itraconazole, and griseofulvin. However, widespread antifungal resistance has emerged, particularly to terbinafine, due to point mutations in the squalene epoxidase (SQLE) gene. A newly recognized species, *Trichophyton indotineae*, associated with genotype VIII of the *T. mentagrophytes*/*interdigitale *complex, has become endemic in India and is linked to high-level terbinafine resistance [6].

Given this backdrop, the present study evaluated the clinico-microbiological profile of dermatophytosis cases. Objectives include assessing clinical patterns, performing potassium hydroxide (KOH) microscopy and fungal culture, and determining the minimum inhibitory concentration (MIC) of antifungals through broth microdilution, with the goal of guiding effective, evidence-based treatment strategies.

## Materials and methods

Study design

A descriptive, cross-sectional study design was adopted.

Study area

The study included clinically diagnosed cases of dermatophytosis and patients with high clinical suspicion of dermatophytosis but negative KOH smear. These patients presented to the Dermatology Outpatient Clinic at Maharishi Markandeshwar Institute of Medical Sciences and Research (MMIMSR), Mullana, Ambala, India. The study was conducted over a 12-month period from March 2024 to March 2025.

Study population and sample size

The study included 130 patients, of either gender, aged above five years. Cases of isolated *Tinea capitis* and *Tinea unguium* (involvement of hair or nails only) and samples showing growth of non-dermatophyte fungi on culture were excluded.

Ethical considerations

Institutional Ethics Committee approval was obtained prior to the commencement of the study (IEC-2940). Patients were enrolled after receiving an in-depth explanation about the nature of the study, and informed consent was obtained, reassuring confidentiality of the information shared.

Study measures

A structured proforma was used to collect data on demographic details, clinical history, previous treatments, hygiene practices, and risk factors. The Dermatophytosis Severity Score (DSS) was calculated based on clinical presentation [7]. Skin scrapings were taken from the active margins of lesions after cleaning with 70% alcohol. Samples underwent KOH mount microscopy and were cultured on Sabouraud dextrose agar (SDA) containing chloramphenicol and cycloheximide. Species identification was confirmed using colony morphology, lactophenol cotton blue (LPCB) mount, dermatophyte test medium (DTM), and urease test. Antifungal susceptibility testing was performed using the CLSI M38-A2 broth microdilution method to determine MICs for oral antifungal agents.

Statistical analysis

All samples were analyzed using Microsoft Excel (Microsoft Corp., Redmond, WA) and SPSS software Version 21 (IBM Corp., Armonk, NY). Proportions and percentages were used as appropriate, depending on the nature of the data obtained for the study. Sensitivity for the antifungal drugs was calculated.

## Results

This study examined 130 patients who met the inclusion and exclusion criteria: 115 patients were KOH positive and 15 were KOH negative. Due to high clinical suspicion, 15 KOH-negative samples (part of inclusion criteria) were also processed for fungal culture, and they came out to be negative. Out of 115 KOH-positive samples, 90 samples had dermatophyte growth on fungal culture, which were further tabulated and processed for antifungal susceptibility testing, and the rest of the samples were discarded.

The most affected age group was 31-45 years (38; 42.2%), followed by 15-30 years (27; 30%). Males (52; 57.8%) were more frequently affected than females (38; 42.2%) (Table 1). A significant 72 (80%) patients were from rural areas. Homemakers (32; 35.6%) and students (19; 21.1%) made up the largest occupational groups, and a majority of patients were uneducated (58; 64.4%).

**Table 1:** Distribution of patients in terms of sociodemographic details

Variable	Domain	Frequency	Percentage
Age	15-30 years	27	30.0%
	31-45 years	38	42.2%
	46-60 years	18	20.0%
	61-75 years	7	7.8%
Gender distribution	Males	52	57.8%
	Females	38	42.2%
Marital status	Married	69	76.7%
	Unmarried	21	23.3%
Locality	Urban	18	20.0%
	Rural	72	80.0%
	Total	90	100.0%

Most patients (40; 44.4%) had a disease duration of one to two years, while 83 (92.2%) had recurrent infections and seven (7.8%) had recalcitrant infections. A large number (79; 87.8%) had mild DSS between 1 and 10. Despite this, recurrent infections were common, even in patients with mild DSS.

**Figure 1:**
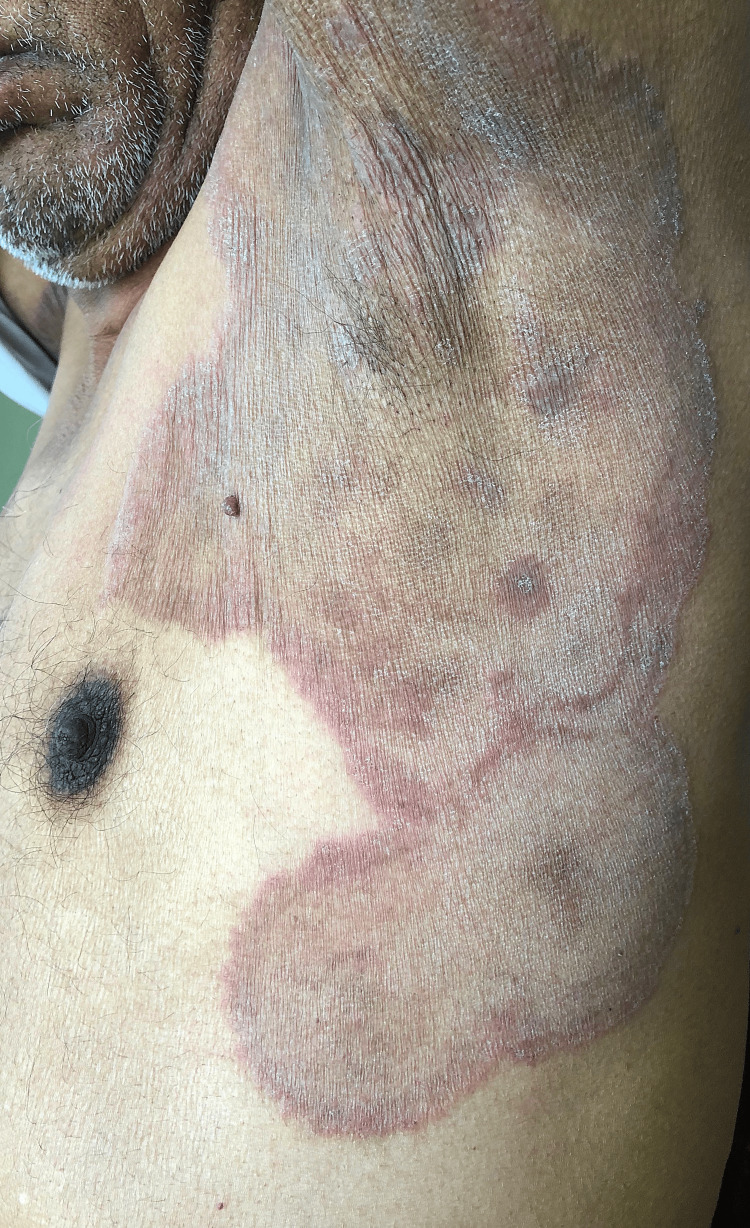
Clinical picture of an extensive Tinea corporis showing multiple erythematous annular scaly plaques with central clearing over the axilla and trunk.

**Figure 2:**
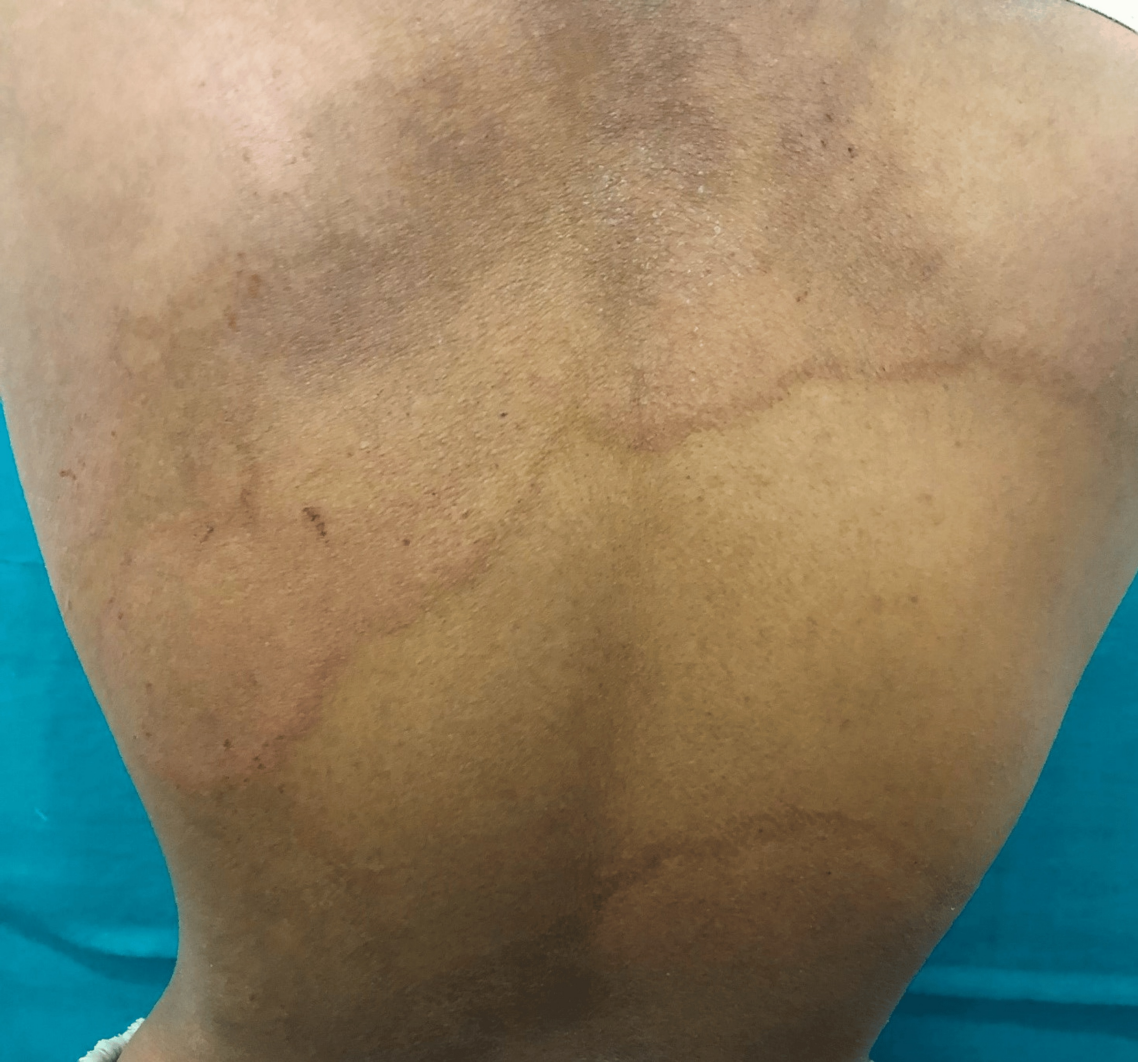
Clinical picture of extensive Tinea corporis showing erythematous annular scaly plaques over the back of the trunk.

*Tinea corporis* (79; 87.8%) (Figures 1, 2) was the most common clinical type, followed by *Tinea cruris* (51; 56.7%) and *Tinea faciei *(20; 22.2%). *Tinea unguium*, though rare (5; 5.6%), was associated with higher DSS. Patients had multiple sites involved, which could lead to generalized tinea. Only 11 (12.2%) patients had a positive family history, mostly with mild disease.

Mild DSS was more common in those with antifungal history, but moderate-to-severe cases were linked to topical steroid misuse (35; 38.9%) and combination creams (10; 11.1%). Alarmingly, 23 (25.6%) patients were unaware of the contents of the creams used, and many sought treatment from pharmacists or unqualified practitioners (35; 38.9%), while only eight (8.9%) patients consulted dermatologists.

Poor hygiene practices had a strong association with disease severity. Reusing unwashed clothes, infrequent bathing with soap, and changing clothes less frequently were all linked to higher DSS.

Diagnosis was primarily done by KOH mount, which was positive in 115 (88.5%) cases. Fungal culture confirmed growth in 95 patients, with *T. mentagrophytes* being the predominant species (87; 75.7%). *Trichophyton rubrum *was rare (3; 2.6%), confirming a shift in the causative organism pattern (Table 2).

**Table 2:** Distribution of patients according to KOH positivity and causative organism KOH, potassium hydroxide

Diagnostic methods		Frequency	Percent
KOH mount	Positive	115	88.5%
	Negative	15	11.5%
Sabouraud dextrose agar culture media of KOH mount positive cases	T. mentagrophytes	87	75.7%
	T. rubrum	3	2.6%
	*Aspergillus* species	5	4.3%
	No growth	20	17.4%
	Total	115	100.0%

Antifungal susceptibility testing revealed high resistance to fluconazole (77; 85.6%) and terbinafine (54; 60%), while no resistance was observed to itraconazole, ketoconazole, and griseofulvin (Table 3).

**Table 3:** Distribution of patients according to drug and their sensitivity

Drug		Frequency	Percent
Fluconazole	Resistant	77	85.6%
	Sensitive	13	14.4%
Terbinafine	Resistant	54	60.0%
	Sensitive	36	40.0%
Itraconazole	Resistant	0	0.0%
	Sensitive	90	100.0%
Ketoconazole	Resistant	0	0.0%
	Sensitive	90	100.0%
Griseofulvin	Resistant	0	0.0%
	Sensitive	90	100.0%
	Total	90	100.0%

## Discussion

Dermatophyte infections remain one of the most common fungal infections seen in dermatology outpatient settings worldwide, with India representing a significant burden due to its climatic conditions and socioeconomic factors. Over recent decades, there has been a noticeable rise in the prevalence, chronicity, and therapeutic challenges posed by dermatophytosis in India. This evolving pattern, characterized by prolonged disease duration, frequent recurrences, and mounting antifungal resistance, signals a shift from what was traditionally considered a simple superficial fungal infection to a chronic dermatological challenge with significant morbidity [8].

Out of 130 patients who met the inclusion and exclusion criteria, 115 patients were KOH positive and 15 were KOH negative. Due to high clinical suspicion, 15 KOH-negative samples (part of inclusion criteria) were also processed for fungal culture, and they came out to be negative. Out of 115 KOH-positive samples, 90 samples had dermatophyte growth on fungal culture, which were processed further for antifungal susceptibility testing, and the rest of the samples were discarded. The present study, including 90 patients with chronic superficial dermatophytosis, provides insight into the demographic, clinical, mycological, and therapeutic aspects of this evolving epidemic in India. The predominance of patients aged 31-45 years (42.2%) followed by those aged 15-30 years (30%) is consistent with other Indian studies such as that by Khurana et al. [9], who documented a peak incidence in the 21-40 years. This trend underscores the vulnerability of the economically productive and socially active segment of the population. Increased outdoor activities, sweating, and close interpersonal contact likely contribute to higher transmission and persistence in this group.

Male predominance (57.8%) aligns with findings reported by Patro et al. [10] and others. The higher incidence among males may be multifactorial, involving greater occupational exposure, increased physical activity, and possibly sociocultural factors influencing healthcare-seeking behavior. Notably, 76.7% of patients in our study were married, suggesting that close family contact and shared personal items such as towels or bedding might facilitate intra-familial transmission. This is compounded by the fact that 80% of patients hailed from rural areas where environmental factors such as humidity, poor sanitation, and limited healthcare access likely exacerbate fungal persistence and spread. Similarly, Kumar et al. [11] reported a significant rural predominance in dermatophytosis cases, highlighting socioenvironmental determinants as key contributors.

Occupationally, homemakers (35.6%) and students (21.1%) formed major proportions. The community environments, such as hostels, and repetitive exposure to fungal reservoirs in households may increase susceptibility in these groups. Farmers and unskilled workers also had notable representation, reflecting the role of prolonged exposure to soil, moisture, and unhygienic working conditions favoring dermatophyte colonization.

Educational status emerged as an important correlate of disease dynamics. A majority (64.4%) of patients were uneducated, paralleling findings by Verma et al. [12] and Shen et al. [13], who linked lower education levels with delayed diagnosis, inadequate treatment adherence, and poor preventive practices. Conversely, those with higher education were less prone to recurrence, likely due to better awareness of hygiene and early medical intervention.

Chronicity was evident, with 44.4% of patients suffering from disease for one to two years and 36.7% for less than a year. Importantly, 92.2% had recurrent dermatophytosis, while 7.8% were recalcitrant cases. This high recurrence rate is in line with national trends, indicating an epidemic-like increase in chronic and resistant infections, particularly involving *T. indotineae* and *T. mentagrophytes*. Verma et al. [12] and Sentamilselvi et al. [14] similarly documented prolonged disease courses and high relapse rates despite treatment. The predominance of disease duration ≤2 years in recalcitrant cases suggests that antifungal resistance and chronicity can manifest early, especially with improper treatment or steroid misuse, as also noted by Rudramurthy et al. [15].

Clinical severity, assessed by the DSS, was predominantly mild to moderate in recurrent cases, indicating that even less extensive disease can relapse repeatedly, possibly due to inadequate therapy, patient non-compliance, or persistent environmental reservoirs. Recalcitrant cases, though fewer, also primarily had low DSS, highlighting that clinical severity does not always correlate with resistance or chronicity. This reinforces the utility of DSS as a clinical tool to guide management, as advocated by Bhat et al. [7].

*Tinea corporis *(87.8%) and *Tinea cruris* (56.7%) were the common clinical forms observed, consistent with regional epidemiology. *Tinea unguium*, though infrequent (5.6%), was associated with higher severity scores, possibly reflecting more chronic, difficult-to-treat infections. Similarly, *Tinea faciei *cases demonstrated moderate-to-severe involvement in a notable fraction, underscoring the potential for extensive disease in certain clinical variants.

Family history was positive in only 12.2% of patients, and it did not significantly influence disease severity. This suggests that environmental exposures, treatment patterns, and personal hygiene play larger roles in disease burden than hereditary or familial predisposition alone.

Drug history revealed that 96.7% of patients had received treatment, with many patients using multiple modalities. Steroid misuse was highly prevalent, with 38.9% patients using topical steroids, 17.8% using antifungal creams alone, and 11.1% using triple combination creams containing steroids, antifungal, and antibiotics. This widespread and irrational use of topical corticosteroids, often obtained over-the-counter or from unqualified practitioners (38.9%), is a well-documented driver of chronicity and resistance in Indian dermatophytosis. Verma et al. [12] and Singh et al. [16] have emphasized the epidemic nature of steroid misuse fueling persistent infections. Our findings that steroid users had higher DSS compared to antifungal-only users further confirm the deleterious impact of inappropriate steroid application.

Hygiene practices were also closely linked with disease severity. Reuse of unwashed clothes (reported by 85.6%) and irregular soap use correlated with higher DSS, highlighting the importance of proper hygiene in disease control. These findings support public health messaging promoting daily changing and washing of clothes and regular bathing with soap to reduce fungal transmission and severity.

For diagnosis, KOH mount proved highly effective with an 88.5% positivity rate, reaffirming its role as a rapid, cost-effective screening tool in resource-limited settings. Culture confirmed the diagnosis in 95 cases (82.6% of positives), with some culture negativity likely due to prior antifungal therapy or low fungal load, as noted by Kidd and Weldhagen [17]. The shift in predominant species from *T. rubrum *to *T. mentagrophytes *seen in this study (75.7% versus 2.6%) aligns with recent Indian data and highlights a changing epidemiological landscape. This shift has important clinical implications given the association of T. mentagrophytes with increased antifungal resistance.

Antifungal susceptibility testing revealed alarming resistance patterns. Fluconazole resistance was highest at 85.6%, paralleling reports by Ebert et al. [6]. Terbinafine resistance was noted in 60% of isolates. This is consistent with Rudramurthy et al.’s findings [15], who linked such resistance to mutations in the squalene epoxidase gene, particularly in *T. mentagrophytes* and *T. interdigitale*. In India, there is a rampant use of fluconazole by unqualified practitioners in subtherapeutic doses. As per verbal communication and records available with the patient, oral fluconazole was given in inappropriate doses and duration, which could have been the reason for resistance in addition to poor patient compliance.

Interestingly, no resistance was observed to itraconazole, ketoconazole, and griseofulvin in our study. This is encouraging and supports their continued use in resistant and chronic cases. Martinez-Rossi et al. [18] highlighted that while resistance to terbinafine and fluconazole is rising, itraconazole remains effective in many cases. The absence of resistance to griseofulvin and ketoconazole may be attributed to their reduced usage in recent years, which could have limited the selection pressure on dermatophytes.

These results emphasize the necessity for routine antifungal susceptibility testing in chronic or recurrent cases to optimize therapy and prevent further resistance development. The widespread misuse of topical steroids and OTC combination creams, particularly in rural areas, underscores the urgent need for regulatory enforcement, healthcare provider education, and public awareness campaigns. Emphasizing rational drug use, discouraging self-medication, and improving hygiene practices should form the cornerstone of intervention strategies.

## Conclusions

The current study reflects the changing epidemiology and clinical profile of dermatophytosis in India, marked by a predominance of *T. mentagrophytes* complex and rising resistance to commonly used antifungals such as fluconazole and terbinafine. Chronicity, recurrence, and steroid misuse contribute significantly to the disease burden. Targeted public health efforts, region-specific treatment guidelines, and incorporation of antifungal susceptibility testing are critical to controlling this emerging dermatological epidemic. Further multicenter studies with larger cohorts and molecular analyses are warranted to better understand resistance mechanisms and effective management strategies.
